# Whole-genome sequencing analysis of phylogenetic relationships and lineage diversity of *Mycobacterium tuberculosis* in Western Kenya

**DOI:** 10.3389/fmicb.2026.1980516

**Published:** 2026-10-09

**Authors:** Susan Musau, Eleanor I. Lamont, Victor Moses Musyoki, Susan Odera, Noel Onyango, Steve Wandiga, Inosters Wambua Nzuki, Bob Morrison, Reymond Mumo, David Sherman, Marianne W. Mureithi, Videlis Nduba

**Affiliations:** 1 Department of Medical Microbiology and Immunology, University of Nairobi, Nairobi, Kenya; 2 Tuberculosis and Human Immunodeficiency Virus (HIV) Co-infection Training Program, Nairobi, Kenya; 3 Department of Microbiology, University of Washington, Seattle, WA, United States; 4 KAVI-Institute of Clinical Research, University of Nairobi, Nairobi, Kenya; 5 Kenya Medical Research Institute, Centre for Global Health Research, Kisumu, Kenya; 6 Center for Biotechnology and Bioinformatics, University of Nairobi, Nairobi, Kenya; 7 Institute of Human Virology (IHV), University of Maryland School of Medicine, Baltimore, MD, United States; 8 Kenya Medical Research Institute, Centre for Respiratory Diseases Research, Nairobi, Kenya

**Keywords:** lineage diversity, *Mycobacterium tuberculosis*, phylogenetics, tuberculosis surveillance, Western Kenya, whole-genome sequencing

## Abstract

**Background:**

Western Kenya remains a high-burden tuberculosis (TB) region, yet genomic data describing the diversity and phylogenetic relationships of *Mycobacterium tuberculosis* complex (MTBC) strains are limited. Understanding sub-lineage patterns and the phylogenetic relationships is essential for effective TB surveillance and control strategies.

**Methods:**

This was a cross-sectional whole genome sequencing (WGS) study using archived MTBC isolates from 12 counties in Western Kenya. Genomic DNA was extracted using a modified CTAB protocol and sequenced using the Element AVITI platform. Reads were processed using DuffyNGS/DuffyTools pipeline and aligned to the H37Rv reference genome. Lineages were assigned using TBProfiler v4.3.0 based on single nucleotide polymorphisms (SNP). Phylogenetic relationships were reconstructed using Nextstrain and interpreted using tree topology.

**Results:**

Of 316 MTBC isolates processed, 290 met quality criteria for WGS. Lineage 4 (L4) (42%) and Lineage 3 (L3) (38%) predominated, with significant county-level heterogeneity observed in Kisii and Homa Bay (L4), and Siaya and Migori (L3) (*p* < 0.0296). Sub-lineage analysis revealed widespread circulation of L2.2.1 and L3.1.1 strains, alongside substantial L4 diversity. Rare sub-lineages showed limited geographic distribution and one *Mycobacterium bovis* isolate was identified. Phylogenetic analysis revealed geographically structured relatedness, with a localized circulation in Kisii, broader regional distribution in Homa Bay, and additional clades showing intermediate regional patterns.

**Conclusion:**

Western Kenya harbors genetically diverse and spatially MTBC population with distinct patterns of phylogenetic relatedness. These findings demonstrate the value of WGS for characterizing population structure and informing targeted TB control in high-burden settings.

## 1 Introduction

Tuberculosis is a disease that can be prevented and is typically curable. However, TB resumed its position as world's leading cause of death from a single infectious agent in 2023 after 3 years of coronavirus disease (COVID-19) predominance (United Nations, 2024). The World Health Organization (WHO) Global TB Report 2024 estimates that 10.8 million people contracted TB in 2023 (up from 10.7 in 2022), with 1.09 million HIV-negative people and 161,000 HIV-positive people dying from the disease (WHO, 2024). Africa carries a significant and disproportionate share of the global tuberculosis burden, with 24% of all cases in 2023 occurring on the continent (WHO, 2024). The high TB burden in Africa highlights the urgent need for strengthened prevention, diagnosis, and treatment efforts.

Kenya is still classified among the 30 high TB burden countries (Enos et al., 2018) with an estimated TB incidence of 223 per 100 000 population (World Health Organization, 2024). The country's estimated TB and Drug-Resistant TB (DR-TB) cases in 2023 were 124,000 and 706, respectively (MOH Kenya, 2023). The genetic diversity of circulating MTBC strains, drug resistance, and continuous transmission all pose challenges to TB control despite tremendous advancements in diagnosis and treatment (Kerubo et al., 2022; Chelimo et al., 2024). In this context, WGS provides high-resolution characterization of strain diversity and phylogenetic clustering consistent with strain relatedness (Meehan et al., 2019; Gordon et al., 2021; Guerra-Assunção et al., 2015).

The MTBC comprises several closely related species, including *M. tuberculosis, M. africanum, M. bovis*, and others, which vary in virulence, transmissibility, and host adaptation (Mvubu and Jacoby, 2023). Although MTBC was once considered genetically uniform, genomic studies have revealed lineage differences that may influence phenotypic traits (Gagneux, 2018; Coscolla et al., 2021). Strain diversity and distribution are critical for TB control because they affect diagnosis, disease outcomes, and drug resistance spread (Osei-Wusu et al., 2022; Yuen et al., 2015). Beyond lineage frequencies, SNP phylogeny distinguishes closely related and more genetically divergent strains (Guerra-Assunção et al., 2015; Wyllie et al., 2018).

In Kenya, the few molecular epidemiological studies have provided insight into MTBC lineages circulating in major urban regions such as Nairobi using both conventional and WGS techniques (Kerubo et al., 2022; Ogaro et al., 2013). Studies in Western Kenya by Chelimo et al. (2024) and Ogwang et al. (2023), showcase TB diversity, using conventional genotyping methods but further characterization was recommended for orphan isolates unresolved by spoligotyping. Therefore, detailed TB genomic data remain limited for Western Kenya, an area characterized by high TB and HIV co-infection rates and cultural diversity (Otiende et al., 2019; Otieno and Okuku, 2017). Importantly, conventional typing lacks sufficient resolution which WGS provides (Wyllie et al., 2018; Chihota et al., 2018).

Determining the diversity of MTBC strains is essential for understanding TB evolution and transmission (Mekonnen et al., 2019). Characterizing genetic diversity and integrating phylogenetic data with geographical metadata reveal spatial distribution and local vs. inter-county transmission dynamics guiding targeted surveillance and interventions in high-burden settings such as Western Kenya (Talarico et al., 2018; Maguga-Phasha et al., 2017; Bakuła et al., 2023).

This study aimed to identify MTBC strains circulating in Western Kenya. It focused on genetic diversity and phylogenetic relationships to contextualize lineage patterns within potential geographic or phylogenetic clustering across 12 counties in Western Kenya.

## 2 Methods

### 2.1 Study design and setting

This was a cross-sectional study involving laboratory analysis of MTBC isolates from TB cases diagnosed in Western Kenya. The study utilized archived isolates obtained from the Kenya Medical Research Institute (KEMRI)/Centre for Global Health Research (CGHR) TB Laboratory biorepository in Kisumu, Kenya. The Laboratory serves as a regional reference facility across 12 counties in Western Kenya—Kisumu, Migori, Siaya, Homa Bay, Kisii, Nyamira, Busia, Kakamega, Vihiga, Bomet, Bungoma, and Kericho for routine diagnostic testing among others. The isolates were obtained from patients with TB-consistent respiratory symptoms in 2021–2022 and evaluated using the Kenya National TB Diagnostic Algorithm.

A county-stratified proportional sampling design was used to select archived MTBC isolates for genomic analysis from the 12 counties in Western Kenya. The sampling frame comprised 1,053 archived isolates from the KEMRI/CGHR TB Laboratory. The isolates were stratified according to county of origin, and proportional allocation was used to determine the initial number of isolates selected from each county to achieve a target sample size of 316.

Eligible isolates included those recovered from Xpert MTB-positive cases, with or without rifampicin resistance; individuals at high risk for drug-resistant TB; special-risk populations; follow-up drug-resistant TB patients; and selected new smear-positive/Xpert-negative cases requiring differentiation of *Mycobacterium tuberculosis* from nontuberculous mycobacteria (NTM). Isolates that were negative on Mycobacteria Growth Indicator Tube (MGIT) culture or contaminated were excluded.

Within each county, non-viable or poor-quality archived isolates were replaced with other eligible isolates from the same county. The selection process also ensured adequate inclusion of isolates with available drug-resistance information, reflecting the characteristics of the archived laboratory collection. Of the 316 isolates processed for WGS, 26 failed the predefined WGS/QC criteria and were excluded. Twelve had nucleic acid concentrations below 50 ng/μL and failed library preparation, while 14 failed sequencing/QC due mainly to inadequate coverage, with some showing evidence of mixed infection. Thus, 290 isolates were retained for analysis (Figure 1).

**Figure 1:**
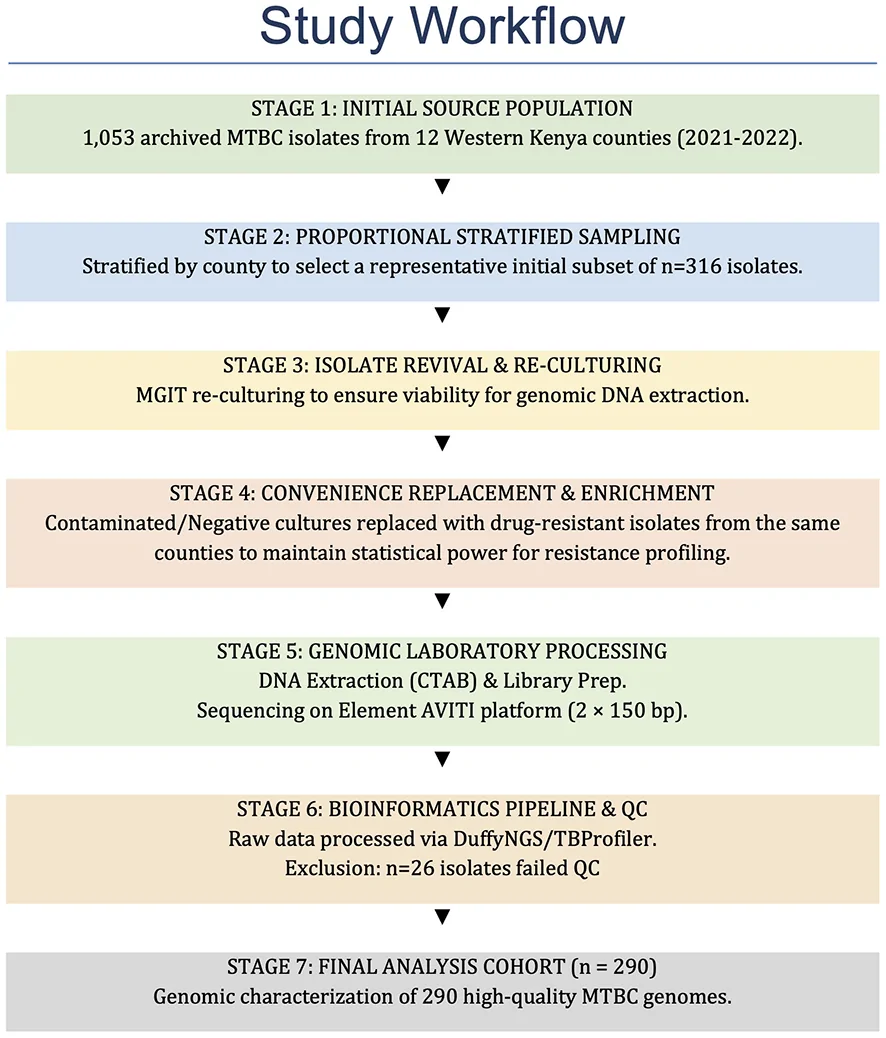
Study workflow and isolate selection for genomic characterization. MTBC, *Mycobacterium tuberculosis* complex; WGS, whole-genome sequencing; MGIT, Mycobacteria growth indicator tube; CTAB, cetyltrimethylammonium bromide; QC, quality control. Selection strategy transitioned from proportional stratified sampling to convenience enrichment to prioritize drug-resistance characterization. The figure has been adapted/revised from the previously published figure, rather than reproduced verbatim. Licensed under Creative Commons Attribution (CC BY). Source: Frontiers in Tuberculosis (Musau et al., 2026).

The unit of selection and analysis was the archived MTBC isolate rather than the individual patient. All eligible archived isolates meeting the study selection criteria were considered irrespective of whether they originated from initial or follow-up testing. No patient-level deduplication or selection of a single representative isolate per patient was performed. Accordingly, the 290 isolates retained after sequencing and bioinformatics quality control were treated as individual isolate genomes in the genomic and phylogenetic analyses.

### 2.2 Laboratory procedures

#### 2.2.1 Sample processing

Sputum specimens were previously decontaminated using the NaOH/N-acetyl-L-Cysteine technique. Following decontamination, a pellet of 0.5 mL was aliquoted in a vial and stored in a negative 80 °C freezer and then cultured on MGIT containing 7 mL of modified Middlebrook7H9 broth base. The medium was enriched with a growth supplement and Polymyxin B, Amphotericin B, Nalidixic Acid, Trimethoprim, and Azlocillin (PANTA) antibiotic included to suppress contamination. Following incubation, the sediment was utilized for identification of MTBC using BD MGIT™ TBc. The Purity of the positive cultures was assessed by inoculating on blood agar or brain heart infusion agar and followed up for any growth after 48 h' incubation. The pellets were subsequently aliquoted into 2 mL cryo-vial for archival.

#### 2.2.2 Genomic DNA extraction

The archived pellets were thawed and inoculated into MGIT culture and growth followed up for MTBC identification before DNA extraction. To maximize DNA yield and purity for WGS, a modified CTAB-based extraction method was used as described by Musau (2026).

#### 2.2.3 Library preparation and whole genome sequencing

Genomic DNA extracted using modified CTAB method was used for WGS after confirming integrity by agarose gel electrophoresis and assessing purity and concentration with NanoDrop spectrophotometry (USA).Genomic libraries were prepared using the NEBNext Ultra II FS DNA Library Preparation Kit (New England Biolabs, USA) following the manufacturer's protocol, then normalized and pooled for sequencing (New England Biolabs, 2023). Pooled libraries were quantified using KAPA Library Quantification Kit (Roche, USA), normalized and sequenced on the Element Biosciences AVITI platform at the Northwest Genomics Center, University of Washington, using paired-end 150-bp (2 × 150 bp) sequencing with read quality reported as Phred >30.

#### 2.2.4 Whole-genome sequencing analysis and lineage assignment

Raw reads generated on the Element AVITI platform were processed using the DuffyNGS/DuffyTools pipeline (v1.8.0; https://github.com/robertdouglasmorrison). Sequence quality was assessed with QuickQC, adapter and low-quality bases were trimmed, and cleaned reads were aligned to the *Mycobacterium tuberculosis* H37Rv reference genome (NC_000962.3) using Bowtie2. Alignment metrics were summarized with Samtools and samples with at least 5 million reads mapped to the *M. tuberculosis* reference genome were retained for downstream analysis. This threshold corresponds to approximately 750 million sequenced bases and a theoretical reference-genome coverage of approximately 170 ×, assuming a 4.4-Mb *M. tuberculosis* genome. The mean number of raw reads generated per sample was approximately 17 million; however, not all raw reads aligned to *M. tuberculosis*.

Samtools/BCFtools algorithms integrated within the DuffyNGS VariantCalls module were used to identify genomic variants, Single Nucleotide Polymorphisms (SNPs) and insertions/deletions (indels).Variant calling was performed using DuffyNGS/DuffyTools v1.8.0 with pipe.VariantCalls(). The documented variant-calling parameters included a minimum read depth of 5 (min.depth = 5), with positions having fewer than five reads not evaluated. The prop.variant = 0.5 parameter was also applied as specified in the variant-calling workflow for SNP retention; according to the laboratory SOP, this parameter determines the threshold for retaining detected SNPs, with higher values retaining more partial SNPs.

Species and lineage assignment was performed from whole-genome sequencing data using TBProfiler v4.3.0. TBProfiler uses a genomic SNP barcode for phylogenetic classification of MTBC isolates, incorporating markers that distinguish *M. tuberculosis* lineages and related MTBC species, including *M. bovis, M. caprae, M. orygis*, and *M. africanum* (Napier et al., 2020).

Lineages were assigned using TBProfiler v4.3.0 based on lineage-defining SNPs and the WHO Catalog of *Mycobacterium tuberculosis* Complex Genetic Mutations Associated with Drug Resistance, version 2. Genetic diversity analyses focused on lineage and sub-lineage distribution, with downstream analyses performed in R (V 4.5.2). Per-sample variant call files (VCFs) were exported for population-level analyses and patients' demographic data were summarized as frequencies and proportions.

#### 2.2.5 VCF analysis and phylogenetics using Nextstrain

VCF-derived SNP data from WGS data were analyzed using the Nextstrain bioinformatics framework (https://nextstrain.org/tb/global) on 21st Feb 2026 using Augur pipeline to reconstruct phylogenetic relationships among the MTBC isolates. SNP sequence data from the sequencing runs were consolidated, reconciled to unique isolates, and formatted as a multiple-sequence FASTA alignment with a common alignment length across runs. Phylogenetic relationships among the 290 *M. tuberculosis* isolates were reconstructed using the Nextstrain/Augur workflow. The final concatenated alignment contained 36,856 nucleotide positions, of which 36,622 were variable, comprising 36,604 parsimony-informative sites and 18 singleton sites; 234 positions were constant. The alignment included genomic regions containing PE/PPE genes, and no additional masking or exclusion of these regions was applied. A maximum-likelihood phylogenetic tree was generated using IQ-TREE under the General Time Reversible (GTR) nucleotide substitution model. The resulting tree was processed using the Augur/TreeTime workflow and visualized in Auspice together with available isolate metadata. The tree was midpoint rooted, and branch lengths represented nucleotide divergence/SNP-scaled evolutionary distance (Walker et al., 2014). No bootstrap replicates or other branch-support analyses were performed. Four major phylogenetic groups were identified descriptively from the topology of the resulting maximum-likelihood tree; these groups were not defined using a predefined pairwise SNP-distance threshold or formal clustering algorithm. Their geographic distributions were subsequently examined using county metadata.

## 3 Ethical considerations

The study utilized de-identified archived isolates and laboratory data with no direct patient contact, ensuring confidentiality. Ethical approval was obtained from the Kenyatta National Hospital-University of Nairobi Ethics and Research Committee (KNH-UoN ERC) and Kenya Medical Research Institute Scientific and Ethics Review Unit (SERU), with additional permission from the National Commission for Science, Technology and Innovation (NACOSTI), KEMRI-CGHR director, and the TB branch head in Kisumu.

## 4 Results

### 4.1 Sociodemographic characteristics of the patients

Among the 290 isolates analyzed, the majority were from male patients (72%), and the median age was 34 years (IQR 26, 43). Individuals aged 25–44 years accounted for the largest proportion (58%), followed by those between 45 and 64 years (17%). 50% of the isolates were from HIV negative patients while 36% were from people living with HIV (Table 1).

**Table 1:** Characteristics of study isolates.

Characteristics	*N* = 290 (%)
Age (years) (IQR)	34 (26–43)
Age category	
0–14	19 (6.6)
15–24	39 (13)
25–44	168 (58)
45–64	49 (17)
65+	15 (5.2)
Gender	
Female	80 (28)
Male	210 (72)
HIV status	
Positive	104 (36)
Negative	145 (50)
Declined/Not done	41 (14)
County	
Bomet	6 (2.1)
Bungoma	6 (2.1)
Busia	9 (3.1)
Homa Bay	56 (19.0)
Kakamega	7 (2.4)
Kericho	2 (0.7)
Kisii	90 (31.0)
Kisumu	13 (4.5)
Migori	31 (11.0)
Nyamira	6 (2.1)
Siaya	32 (11.0)
Vihiga	32 (11.0)

### 4.2 Isolate characteristics

Of the 316 isolates, 290 were successfully sequenced with Kisii County contributing the largest proportion (*n* = 90, 31%), followed by Homa Bay (*n* = 56, 19%) with moderate contributions from Siaya (*n* = 32, 11%), Vihiga (*n* = 32, 11%), and Migori (*n* = 31, 11%). The remaining counties each contributed < 5% of isolates, including Kisumu (*n* = 13, 4.5%), Busia (*n* = 9, 3.1%), Bomet, Bungoma, and Nyamira (*n* = 6 each, 2.1%), Kakamega (*n* = 7, 2.4%), and Kericho (*n* = 2, 0.7%).

L4 was the most predominant lineage (*n* = 121, 41.7%), followed by L3 (*n* = 109, 37.6%) and L2 (*n* = 58, 20.0%). L1 and *M. bovis* were each detected only rarely, with one isolate each (0.3%; Figure 2). Overall, Lineages 3 and 4 together accounted for nearly four-fifths (79.3%) of all isolates, indicating their dominant circulation in Western Kenya.

**Figure 2:**
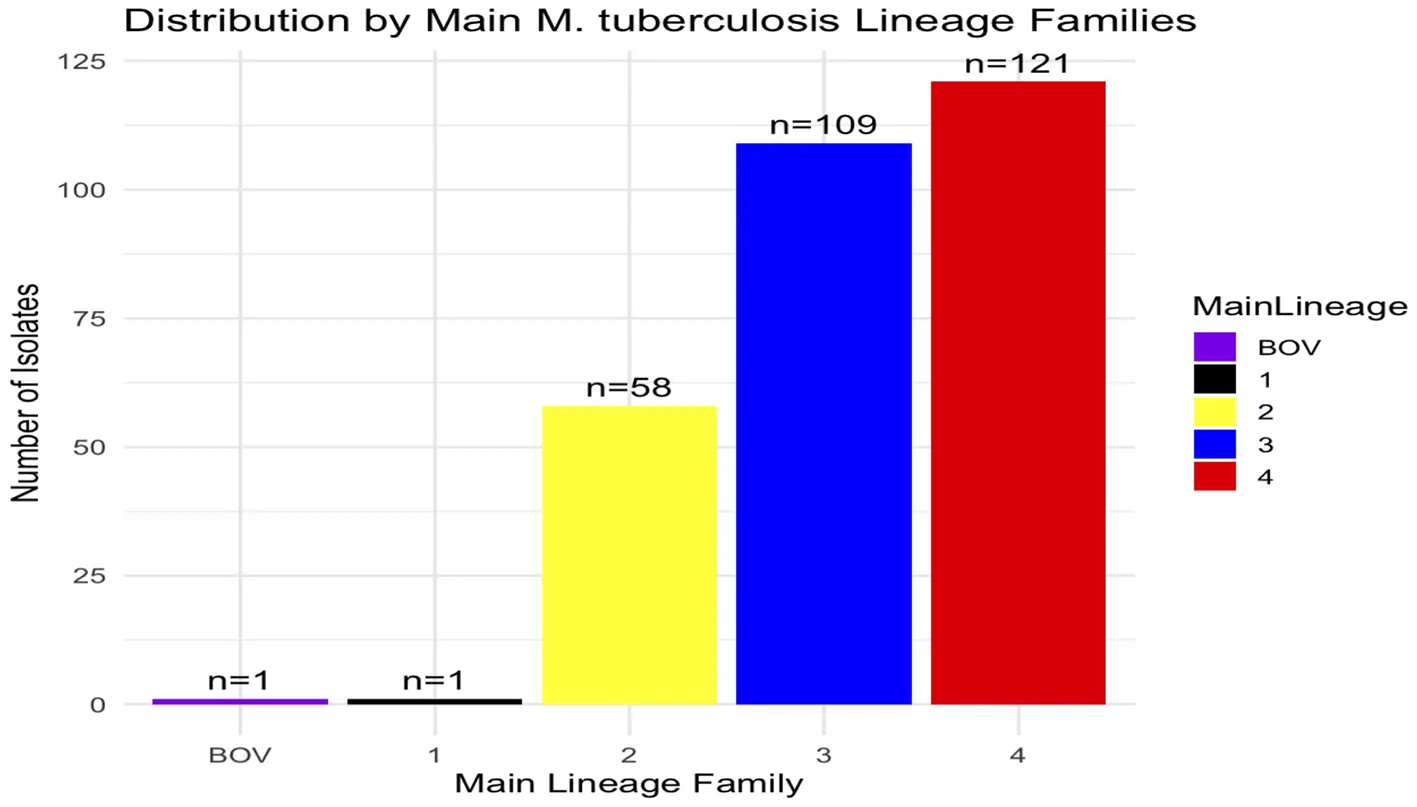
Distribution of MTBC isolates by main lineage family, showing dominance of Lineage 4 and Lineage 3, with limited representation of Lineage 2 and rare occurrence of Lineage 1 and *Mycobacterium bovis*.

L2.2.1 was the most frequent sub-lineage, followed by L3.1.1 confirming the substantial contribution of both historical Beijing and CAS families (Kerubo et al., 2022). Within Lineage 4, multiple sub-lineages were observed, with L4.4.1.1 and L4.3.4.2 contributing the highest numbers, alongside representation of Lineage 4.8, 4.3.3, and 4.2.2. Several regionally associated sub-lineages, including Lineage 4.6.1.2 and 4.6.1.1 historically known as Uganda I and II (Kerubo et al., 2022), were also detected, while other sub-lineages such as 4.6.2, 4.1.2, 4.1.1.3, 4.1.1.1, and Lineage 1.1.2 occurred at low frequencies (Figure 3 and Table 2).

**Figure 3:**
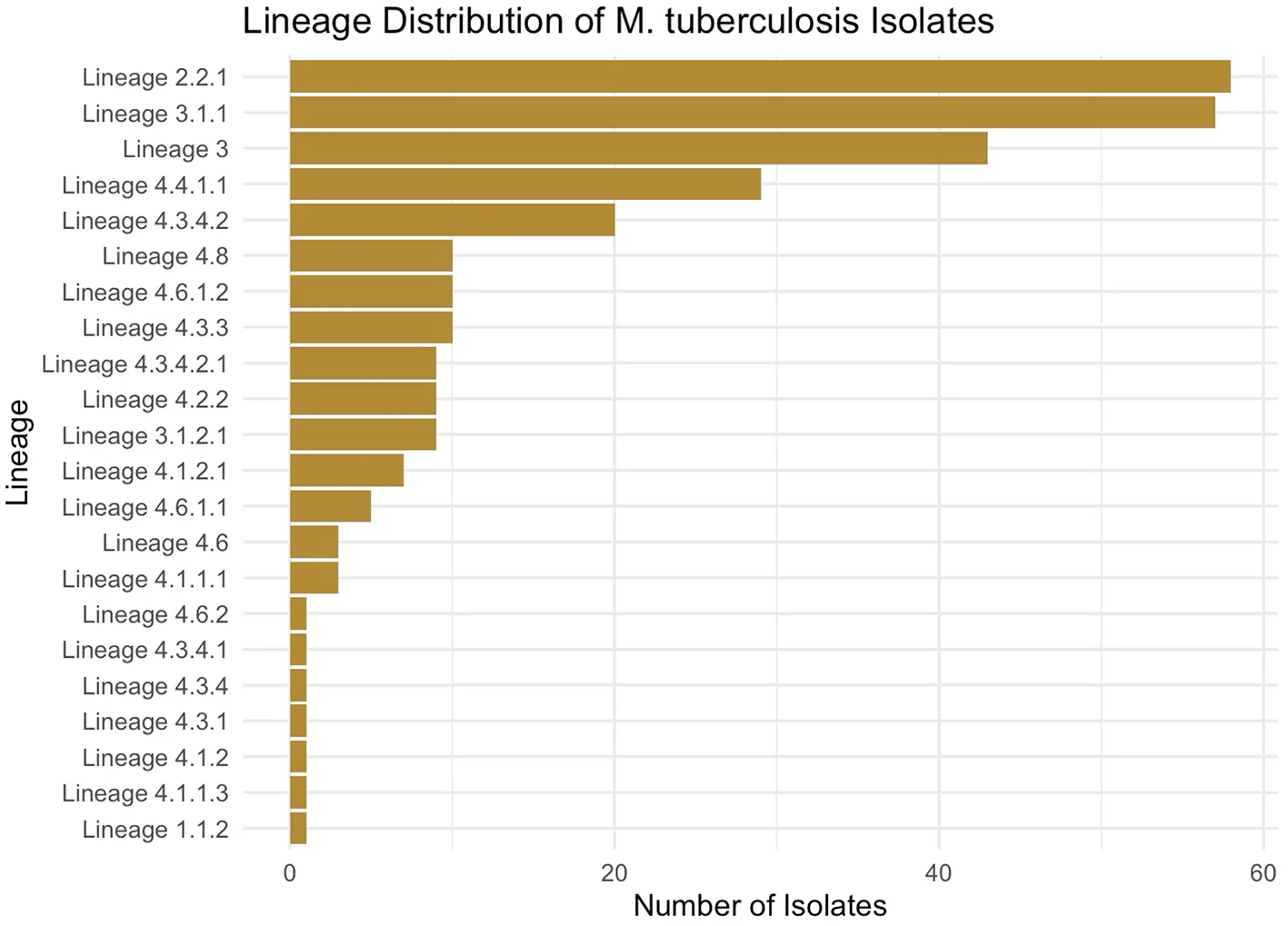
Distribution of MTBC isolates by lineage and sub-lineage, showing predominance of Beijing (Lineage 2.2.1), CAS (Lineage 3), and multiple Lineage 4 families, reflecting high genetic diversity among isolates from Western Kenya.

**Table 2:** Distribution of *Mycobacterium tuberculosis* complex lineages and sub-lineages among isolates from Western Kenya.

Lineage	Sub-lineage	No. of isolates
L1	L1.1.2	1
L2	L2.2.1	58
L3	L3 (not further subtyped)	43
L3	L3.1.1	57
L3	L3.1.2.1	9
L4	L4.1.1.1	3
L4	L4.1.1.3	1
L4	L4.1.2	1
L4	L4.1.2.1	7
L4	L4.2.2	9
L4	L4.3.1	1
L4	L4.3.3	10
L4	L4.3.4	1
L4	L4.3.4.1	1
L4	L4.3.4.2	20
L4	L4.3.4.2.1	9
L4	L4.4.1.1	29
L4	L4.6	3
L4	L4.6.1.1	5
L4	L4.6.1.2	10
L4	L4.6.2	1
L4	L4.8	10
BOV	BOV.	1
Total		290

### 4.3 Sociodemographic and clinical distribution of MTBC lineages

Across the study population, Lineages 3 and 4 predominated across all demographic strata, with consistent co-circulation observed by age, gender, and HIV status. The 25–44-year age group, which contributed the highest number of isolates, showed predominance of L4 (43.5%), followed by L3 (35.1%) and L2 (20.8%); *M. bovis* isolate (0.6%) was identified in this age group. Males contributed a larger proportion of isolates across all major lineages, particularly L4 (*n* = 90) and L3 (*n* = 77), whereas among females, Lineages 3 (*n* = 32) and 4 (*n* = 31) occurred at comparable frequencies, followed by L2 (*n* = 16); the Lineage 1 isolate was detected among females. Among HIV-negative individuals, L3 was most frequent (*n* = 67), followed by L4 (*n* = 52) and L2 (*n* = 26). In contrast, among HIV-positive individuals, L4 predominated (*n* = 50), followed by L3 (*n* = 32) and L2 (*n* = 21); L1 isolate was also identified in this group. *M. bovis* isolate was detected among individuals who declined HIV testing.

### 4.4 County-specific MTBC lineage distribution

Heterogeneity in lineage distribution was observed across the 12 counties in Western Kenya (*n* = 290). L4 was the dominant lineage in high-burden counties, including Kisii (47/90; 52%) and Homa Bay (25/56; 44.6%). L3 was dominant in Siaya (18/32; 56.3%) and Migori (13/31; 41.9%), while contributing substantially in Kisii (30/90; 33.3%) and Homa Bay (20/56; 35.7%). L2 had notable phylogenetic grouping in Migori (10/31; 32.3%), Vihiga (9/32; 28.1%), Homa Bay (11/56; 19.6%) and Kisii (13 /90; 14.4%; Table 3). A Monte Carlo-simulated chi-square showed a significant association between MTBC lineage and county of isolation (χ^2^ =102.08, *p*-value = 0.0296). Cramér's *V* was calculated to quantify the strength of the global association, resulting to a value of 0.297, indicative of a medium effect size.

**Table 3:** County-lineage distribution.

Lineages	Western Kenya counties												Total (%)
	Kisii	Homa Bay	Siaya	Vihiga	Migori	Kisumu	Busia	Kakamega	Bomet	Bungoma	Nyamira	Kericho	
Lineage 1							1						1 (0.3)
Lineage 2	13	11	5	9	11	5	1		2	1			58 (20)
Lineage 3	30	20	18	11	13	2	2	5	1	2	4	1	109 (37.6)
Lineage 4	47	25	9	12	7	6	5	2	3	2	2	1	121 (41.7)
Lineage BOV										1			1 (0.3)
Total	90	56	32	32	31	13	9	7	6	6	6	2	290

Monte Carlo-simulated chi-square (based on 10,000 replicates): χ^2^ = 102.08, *p*-value = 0.0296; Cramér's *V* = 0.297.

### 4.5 County-level distribution of MTBC sub-lineages

Kisii County exhibited the greatest sub-lineage diversity, contributing a large proportion of L2.2.1 (14/58; 24.1%) and L3.1.1 (19/57; 33.3%) strains, alongside substantial representation of L4 sub-lineages, including L4.4.1.1 (16/29; 55%), L4.3.4.2 (10/20; 50%), L4.8 (5/10; 50%), and L4.3.3 (6/10; 60%). Homa Bay County showed a similarly heterogeneous profile, with substantial contributions to L2.2.1, L3.1.1, and L3 (each 19%), together with multiple L4 sub-lineages, including Uganda-associated strains. Siaya contributed of L3 (21%) isolates, while Migori contributed 17% of L2.2.1 isolates. Vihiga County showed substantial contributions to L2.2.1 (17%) and L4.4.1.1, alongside L3 isolates. Rare sub-lineages, including Lineage 1.1.2, Lineage 4.6, Lineage 4.6.2, Lineage 4.1.1.3, and Lineage 4.1.2, were detected at very low frequencies and were confined to single counties (Table 4).

**Table 4:** County-level distribution of *Mycobacterium tuberculosis* complex sub-lineages among the 290 sequenced isolates from Western Kenya.

County	L2.2.1	L3.1.1	L3	L4.4.1.1	L4.3.4.2	L4.8	L4.3.3	L4.6.1.2	L4.2.2	L3.1.2.1	L4.3.4.2.1	L4.1.2.1	L4.6.1.1	L4.1.1.1	L4.6	L4.3.1	LB	L1.1.2	L4.3.4.1	L4.3.4	L4.6.2	L4.1.1.3	L4.1.2
Bomet	2	1	0	0	0	0	0	0	1	0	0	0	0	1	0	1	0	0	0	0	0	0	0
Bungoma	1	1	1	0	0	1	0	0	0	0	0	0	0	1	0	0	1	0	0	0	0	0	0
Busia	1	0	2	0	2	0	0	0	0	0	0	2	1	0	0	0	0	1	0	0	0	0	0
Homa Bay	11	11	8	3	3	1	1	4	3	1	4	2	2	1	1	0	0	0	1	0	0	0	0
Kakamega	0	3	2	0	1	0	0	0	0	0	1	0	0	0	0	0	0	0	0	0	0	0	0
Kericho	0	1	0	0	0	0	0	1	0	0	0	0	0	0	0	0	0	0	0	0	0	0	0
Kisii	14	19	8	16	10	5	6	2	2	4	2	2	1	0	0	0	0	0	0	0	0	0	0
Kisumu	3	1	1	1	2	1	0	0	0	0	1	0	1	0	0	0	0	0	0	0	0	0	0
Migori	10	6	7	2	0	2	0	2	0	0	0	1	0	0	1	0	0	0	0	0	0	0	0
Nyamira	0	2	2	0	0	0	0	0	0	0	0	0	0	0	0	0	0	0	0	1	1	0	0
Siaya	6	7	9	1	0	0	3	1	2	1	1	0	0	0	1	0	0	0	0	0	0	0	0
Vihiga	10	5	3	6	2	0	0	0	1	3	0	0	0	0	0	0	0	0	0	0	0	1	1
Total lineages	58	57	43	29	20	10	10	10	9	9	9	7	5	3	3	1	1	1	1	1	1	1	1

### 4.6 Phylogenetic structure of the MTBC population

Phylogenetic reconstruction of the 290 MTBC isolates revealed substantial genetic diversity structured into four major clades with distinct geographic composition (Figure 4). Clade I (*n* = 74) was dominated by Vihiga (*n* = 29) and Siaya (*n* = 27), with additional isolates from Bomet (*n* = 6), Bungoma (*n* = 4), Nyamira (*n* = 4), Busia (*n* = 2), Migori (*n* = 1), and Kisumu (*n* = 1). Clade II (*n* = 84) was almost entirely restricted to Kisii County (*n* = 82), with only one isolate each from Vihiga and Siaya. Clade III (*n* = 78) was centered in Homa Bay (*n* = 54), with further representation from Busia (*n* = 7), Kakamega (*n* = 7), Siaya (*n* = 4), Nyamira (*n* = 2), Kericho (*n* = 2), Kisii (*n* = 1), and Kisumu (*n* = 1). Clade IV (*n* = 54) was dominated by Migori (*n* = 29), followed by Kisumu (*n* = 12) and Kisii (*n* = 7), with smaller contributions from Homa Bay (*n* = 2), Bungoma (*n* = 2), and Vihiga (*n* = 2).

**Figure 4:**
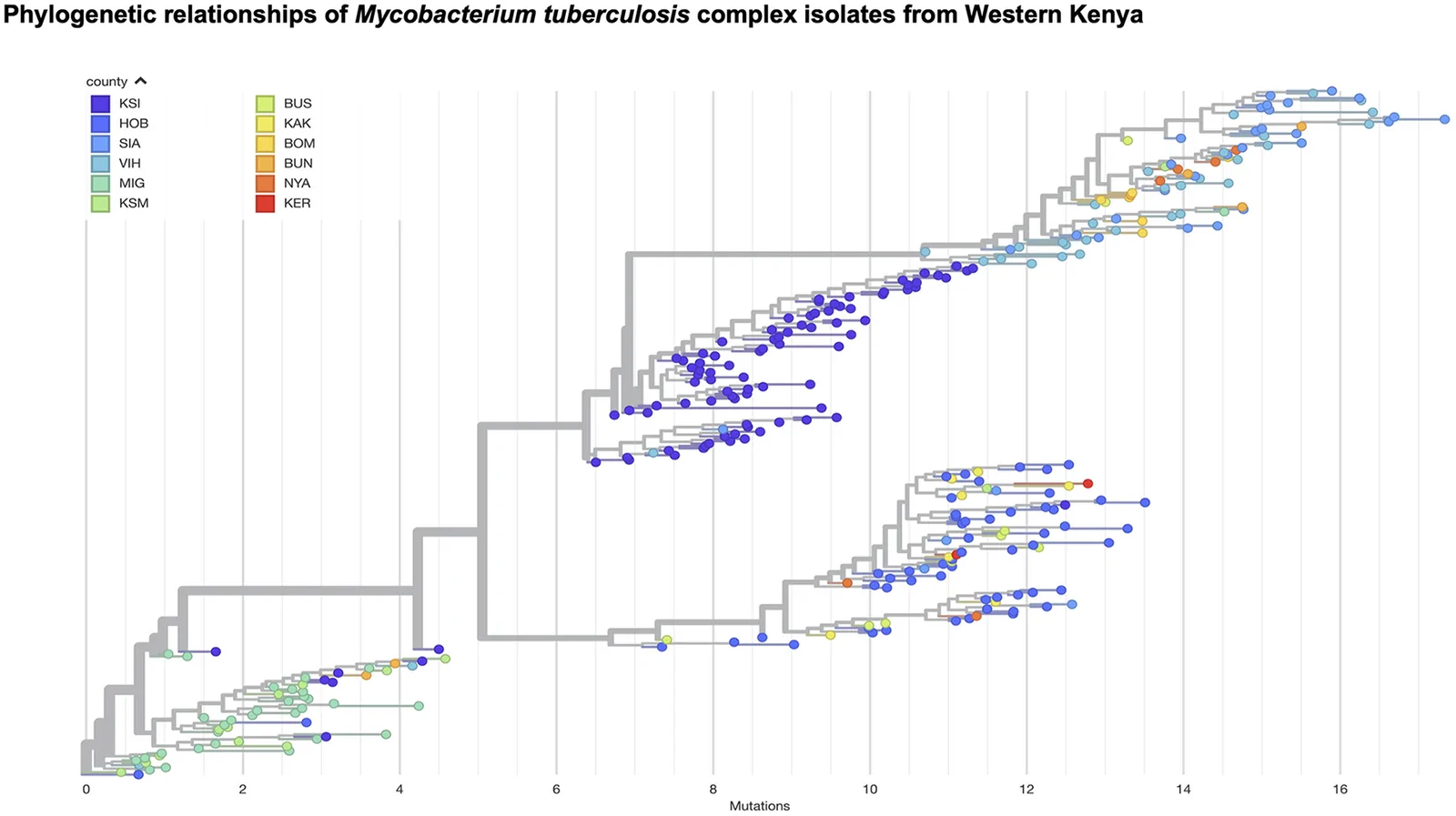
Maximum-likelihood phylogenetic tree of the 290 *M. tuberculosis* isolates. The tree was reconstructed using IQ-TREE under the GTR nucleotide substitution model through the Nextstrain/Augur workflow. The horizontal scale represents SNP-scaled phylogenetic branch length and nucleotide divergence along individual branches; it does not represent absolute pairwise genome-wide SNP differences between isolates.

## 5 Discussion

The genetic diversity of circulating MTBC strains and ongoing transmission continue to challenge TB control despite tremendous advancements in diagnosis and treatment. This necessitates effective TB surveillance and control strategies. This study aimed at identifying MTBC strains circulating in Western Kenya focusing on phylogenetic relationships and genetic diversity in relation to TB control. L4 was the most prevalent, followed by L3. Sub-Lineages 2.2.1 and 3.1.1 were the most frequent. The phylogenetic analysis revealed geographic structuring of MTBC isolates across western Kenya, with localized phylogenetic relatedness among isolates from Kisii, broader regional genetic relatedness involving isolates from Homa Bay, and additional phylogenetic relatedness among isolates from Vihiga–Siaya and Migori.

The lineage distribution in this study aligns with the global epidemiological patterns in which L4 is the most widely distributed MTBC lineage, with L2 contributing a substantial proportion of cases (Stucki et al., 2016; Alvarez-Maya et al., 2025; Jaemsai et al., 2025). Similar trends are reported Africa and East Africa, where L4 predominates while L3 remains highly prevalent, followed by L2, reflecting historical migration and regional connectivity (Chihota et al., 2018; Shitikov et al., 2024). Comparable patterns have been reported in Nairobi (Kerubo et al., 2022) and the near co-dominance of L4 and L3 observed in Western Kenya (Ogwang et al., 2023) highlighting pronounced intra-country heterogeneity. The predominance of Modern Beijing (Lineage 2.2.1) and the broad sub-lineage diversity within Lineage 4, suggest sustained circulation of diverse MTBC lineages in Western Kenya, with rare sub-lineages and *M bovis* isolate reflecting sporadic occurrence reported elsewhere in Africa (Kerubo et al., 2022; Coscolla et al., 2021). Studies conducted in Pakistan and Ethiopia however showed dominance of L3 followed by similar patterns for other lineages (Khan et al., 2025; Ejo et al., 2021). The global dominance of Lineage 4 likely reflects its broad host adaptability, efficient transmissibility, and historical dissemination through human migration, urbanization, and colonial expansion (Gagneux, 2018; Stucki et al., 2016).

The phylogenetic structure observed in this study demonstrates marked geographic stratification of MTBC populations across western Kenya, with distinct patterns of phylogenetic grouping and circulation across counties (Bakuła et al., 2023). Similar geographic and phylogenetic heterogeneity has been described across Africa, where the distribution of MTBC lineages and sub lineages varies across settings and may be influenced by population mobility, and healthcare access particularly within Lineages 3 and 4 (Sahal et al., 2023). The Kisii-dominated clade showed minimal geographic mixing, indicating a concentration of closely related MTBC isolates within the county. This pattern is consistent with localized circulation of related strains, although recent transmission cannot be inferred from phylogenetic topology alone (KNBS, 2019; Omollo et al., 2018). In contrast, the Homa Bay–centered clade displayed broad multi-county representation, suggesting that related MTBC strains circulate across multiple counties in the region. The observed geographic distribution may indicate connectivity between Homa Bay and surrounding areas, although the available phylogenetic analysis does not establish Homa Bay as a source of strain dissemination. This pattern is consistent with the Lake Victoria basin's fishing activities, high disease burden and elevated HIV prevalence, factors known to facilitate TB transmission and wider dissemination of circulating strains (Kwena et al., 2019). The observed grouing may reflect shared ancestry and/or circulation of related strains within and across counties, but that confirmation of recent transmission would require additional genomic analyses, including pairwise SNP-distance assessment, together with epidemiological and temporal linkage data. Together, these findings are consistent with global and regional observations while highlighting substantial geographic variation in the phylogenetic distribution and circulation of MTBC strains within Kenya.

## 6 Limitations of the study

Although stratified sampling improved county representation, the study relied on laboratory-derived archived isolates rather than a population-based sample, with limited clinical and epidemiological data. The inclusion of selected clinical groups and isolates with specific diagnostic and drug-resistance characteristics, together with small sample sizes in some counties and incomplete sampling, may limit representativeness and precision. Consequently, lineage frequencies should not be interpreted as population-level estimates. Because selection and analysis were performed at the isolate level without patient-level deduplication, multiple isolates from the same patient cannot be excluded and may influence lineage frequencies and phylogenetic patterns.

The cross-sectional design and lack of epidemiological linkage data prevented inference of transmission timing or direction. Furthermore, no predefined SNP-distance threshold was used to define genomic clusters. Thus, phylogenetic proximity should not be interpreted as definitive evidence of recent or epidemiologically linked transmission; observed grouping instead reflects genetic relatedness among isolates within the study collection.

## 7 Conclusion

This study demonstrates that Western Kenya harbors a diverse and heterogeneous population of *M. tuberculosis* complex strains, dominated by Lineages 4 and 3 with substantial sub-lineage diversity, including widespread circulation of L2 lineages. The phylogenetic evidence of localized grouping and inter-county circulation of related MTBC strains highlights substantial geographic heterogeneity in the distribution of MTBC in Western Kenya and underscores the value of geographically informed genomic surveillance for understanding strain circulation and informing targeted TB control strategies in high-burden settings.

## 8 Recommendations

These findings support integrating WGS into routine TB surveillance in Kenya. Future studies combining genomic and epidemiological data with improved geographic sampling will strengthen transmission analysis and guide targeted control in high-burden regions such as Western Kenya.

## Data Availability

The datasets presented in this study can be found in online repositories. The names of the repository/repositories and accession number(s) can be found at: https://www.ncbi.nlm.nih.gov/, PRJNA1462751.
